# Can impulsivity evolve in response to childhood environmental harshness?

**DOI:** 10.1017/ehs.2022.22

**Published:** 2022-05-24

**Authors:** Atsushi Kometani, Yohsuke Ohtsubo

**Affiliations:** 1 Department of Psychology, Graduate School of Humanities, Kobe University, Kobe, Japan; 2 Department of Social Psychology, Graduate School of Humanities and Sociology, University of Tokyo, 7-3-1 Hongo, Bunkyo-ku, Tokyo, 113-0033, Japan

**Keywords:** Impulsivity, childhood socioeconomic status, phenotypic plasticity, life history theory

## Abstract

Previous studies have suggested that human impulsivity is an adaptive response to childhood environmental harshness: individuals from families of low socioeconomic status (SES) tend to be more impulsive. However, no studies have tested the evolvability of this reaction norm. This study examined whether (a) impulsivity is associated with higher fitness among individuals from low SES families, while (b) it is associated with lower fitness among individuals from high SES families. We assessed three indices of impulsivity (temporal discounting, risk taking and fast/slow life history strategy), childhood SES and five proxy indices of fitness (number of children, lifelong singlehood, annual household income, subjective SES and life satisfaction) of 692 middle-aged participants (40–45 years old). None of the results supported the evolvability of the impulsivity reaction norm, although low childhood SES was associated with lower fitness on every proxy measure. Impulsivity (operationalised as the fast life history strategy) was associated with lower fitness regardless of childhood SES.

## Graphical abstract

**Figure:**
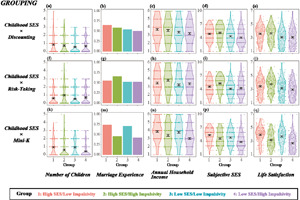

**Social media summary:** Impulsivity is not an adaptive response to childhood environmental harshness among Japanese middle-aged adults.

## Introduction

Phenotypic plasticity has long been recognised but has remained underappreciated until recently in evolutionary biology (Sommer, 2020). Although many instances of plasticity may be by-products of physical and chemical developmental processes, certain kinds of plasticity are adaptive reactions to environmental variation (Nijhout, 2003). Recently, psychological studies on phenotypic plasticity have increased under the rubric of life history theory (Del Giudice et al., 2015; Nettle & Frankenhuis, 2020). However, phenotypic plasticity and life history theory do not necessarily go together in evolutionary biology. Consequently, less attention has been paid to the literature on biological phenotypic plasticity in evaluating purported phenotypic plasticity in humans. In this study, drawing on a biological model of phenotypic plasticity, we critically examine whether an oft-cited human phenotypic plasticity (i.e. early-life adversity causing impulsivity in adulthood) is an evolvable reaction norm.

## Low socioeconomic status and impulsivity

According to Nettle and Frankenhuis (2020), the core idea of life history theory in evolutionary biology is that natural selection acts on phenotypes associated with different components of fitness (e.g. survival, reproduction). Its goal is to understand the population-specific optimisation of trade-offs between different fitness components under different environments. Hence, it is often used to explain across-species/population differences. In contrast, life history theory in psychology almost exclusively focuses on individual differences along the so-called fast–slow continuum: whether each individual prioritises reproduction (leaning towards fast strategies) or survival (leaning towards slow strategies) in response to their local environment.

Such an empirical focus of psychological life history theory has its roots in abundant evidence that low socioeconomic status (SES) is associated with a set of apparently problematic behaviours (or behavioural constellation of deprivation, BCD), such as saving less for the future, having teen pregnancies, investing less in their children, having poorer diets, using illicit drugs, consuming an excessive amount of alcohol and smoking more (Pepper & Nettle, 2017). Although BCD is often viewed as maladaptive, Pepper and Nettle maintain that BCD may be more accurately understood as contextually appropriate responses to environmental uncertainty experienced by low SES individuals (e.g. if you do not expect to survive until next year, there is little point in saving for the distant future). Accordingly, Pepper and Nettle consider that BCD may represent evolved responses to environmental uncertainty.

In addition, life history theory in psychology tends to emphasise the role of childhood environments (Nettle & Frankenhuis, 2020). Its tenet is that early-life adversity, such as childhood economic harshness, causes accelerated reproductive timing and other traits associated with BCD. Empirical studies have supported the effects of early-life adversity (e.g. Griskevicius et al., 2013; Mittal & Griskevicius, 2014). For example, Griskevicius et al. (2013) exposed half of the participants to a series of images indicative of economic recession (recession condition), while the other half was exposed to a series of images of nature (control condition). Participants then engaged in two tasks relevant to BCD (i.e. temporal discounting and risk taking), which Griskevicius et al. referred to as ‘impulsivity’. In the present article, we follow this terminology and use a loose definition of *impulsivity* that refers to a group of traits that are theorised as outcomes of childhood adversity. Dividing participants into high vs. low childhood SES groups by the criterion of one standard deviation (SD) above or below the mean, Griskevicius et al. found a 2 (childhood SES) × 2 (economic threat priming) interaction effect on impulsivity. In the recession condition, individuals from low SES families exhibited greater temporal discounting and risk-taking tendencies than individuals from high SES families, but no such difference was observed in the control condition. If we assume that an immediate economic threat reveals one's disposition, the result is congruent with life history theory in psychology, which predicts that individuals grow more impulsive in response to early-life adversity (but see Amir et al., 2018, for a replication failure).

## Evolution of reaction norms

Although the results of Griskevicius et al. (2013) and other similar findings (see Del Giudice et al., 2015, for a review) are congruent with life history theory in psychology, the psychology version of life history theory has been criticised in various aspects. Critics question, for example, whether there are, in fact, trade-offs in reproduction and survival in humans and whether various traits necessarily cluster together along the fast–slow continuum (see articles in the special issue of *Evolution and Human Behavior* on ‘current debates in human life history research’ edited by Frankenhuis & Nettle, 2020). Although many criticisms have been directed at its validity as a branch of life history theory, some have paid critical attention to another aspect: an evolutionary account of human plasticity (e.g. Galipaud & Kokko, 2020; Nettle et al., 2013).

For example, it may not be adaptive for any long-lived species, such as humans, to set their phenotypes based on their childhood environments because their environments may change over their long lifespans (Nettle et al., 2013). In this case, reversible lifelong plasticity may be more adaptive (Ratikainen & Kokko, 2019). However, there are also reasons to expect that childhood SES may be predictive of one's later environments (Nettle et al., 2013). For example, early-life malnutrition may have lasting effects on one's health status. Educational disadvantage during childhood may also accentuate initial disparities later in life. Although such prolonged effects of early-life adversity may make accelerated reproductive timing adaptive, a non-adaptationist account is also possible: detrimental psychological effects of early-life adversity may be a by-product of disruption of normal brain development (e.g. McLaughlin et al., 2014). Therefore, no firm answer exists for the question of whether apparent human phenotypic plasticity is in fact an instance of an evolved reaction norm.

As a first step to answering this question, we drew on a mathematical model of adaptive reaction norms developed in biology (Hazel et al., 1990; see also Kokko, 2007, for a simplified version). One application of this model is the predator-induced polymorphism in the acorn barnacle *Chthamalus anisopoma* (Lively, 1986a, b). Although they normally develop conical shells, some develop bent shells in the presence of the predatory snail *Acanthina angelica*. This predator-induced polymorphism is adaptive because the bent morph is costly (i.e. less fecund), but more resistant to predators’ attacks. This logic can be readily applied to the impulsivity reaction norm (Table 1). In the absence of childhood environmental harshness (high SES, conceptually corresponding to predator-absence), impulsivity is costly: high impulsivity is associated with lower fitness (*α*_0_) than low impulsivity (*β*_0_) is. In the presence of childhood environmental harshness (low SES, corresponding to predator presence), impulsivity confers fitness benefits: high impulsivity is associated with higher fitness (*α*_1_) than low impulsivity (*β*_1_) is. In addition, it is plausible to assume that environmental harshness adversely influences fitness. Thus, the inequality *β*_0_ > *α*_0_ > *α*_1_ > *β*_1_ must hold for the reaction norm of impulsivity to be evolvable.

**Table 1.** Four types of individuals (groups 1–4) divided by childhood environment (harsh vs. not harsh) and impulsivity (high vs. low)

		Childhood SES	
		High (0)	Low (1)
Impulsivity	High (*α*)	*α*_0_ (group 2)	*α*_1_ (group 4)
	Low (*β*)	*β*_0_ (group 1)	*β*_1_ (group 3)

*Note*: the underline indicates greater fitness in each column.

## The present study

To test whether the above inequality holds, we conducted a modified replication of Griskevicius et al. (2013). In addition to assessing each participant's childhood SES and their impulsivity, we had participants report five proxy indices of fitness (i.e. number of children, marriage experience, annual household income, subjective SES, life satisfaction). We specifically recruited participants in their early 40s because our indices of fitness included the number of children. The set of three measures allowed us to test the aforementioned inequality. The first two measures, which were independent variables, were used to divide participants into four groups: high childhood SES individuals exhibiting low impulsivity (group 1); high childhood SES individuals exhibiting high impulsivity (group 2); low childhood SES individuals exhibiting low impulsivity (group 3); and low childhood SES individuals exhibiting high impulsivity (group 4). We then tested whether the inequality held for each of the five proxy indices of fitness. In particular, we tested whether impulsivity was associated with higher fitness among individuals from low SES families (*α*_1_ > *β*_1_) but lower fitness among individuals from high SES families (*β*_0_ > *α*_0_).

This study has three features that merit additional explanation. First, the proxy indices of fitness included variables that are not usually considered relevant to biological fitness. The straightforward index of fitness is number of children. However, owing to the availability of contraceptive methods in modern environments, successful individuals may not necessarily have more children (e.g. Pérusse, 1993; Vining, 1986; but see Stulp & Barrett, 2016, for counterevidence). Accordingly, we assessed participants’ marriage experiences, which would have led to having children in ancestral environments. Financial success, which is associated with high status, would also have resulted in more children and enable greater parental investment in offspring in ancestral environments. Therefore, we measured participants’ annual incomes and subjective evaluations of their SES. In addition, the overall evaluation of one's quality of life may reflect some components that would have been associated with fitness in ancestral environments. In summary, to fill the gap between modern and ancestral environments, we included a wide range of variables, but recognised that some may not be relevant to fitness.

Second, unlike Griskevicius et al.'s (2013) study, we did not include economic recession priming. Our study was conducted in the midst of the COVID-19 pandemic, and people were continually exposed to real-world news about economic crises. Instead of including the priming task, we assessed whether participants’ incomes had decreased from the year 2019 (one year before data collection and the COVID-19 pandemic) to 2020. Assuming that a real decrease in annual income would have a greater psychological effect than priming tasks, we separately analysed the data from participants whose incomes had decreased from the previous year. However, the analyses of this subset of the sample did not change the general pattern of the results. Therefore, we report the results based on this subsample in the Supplementary Material.

Third, as an additional measure of impulsivity, as it was loosely defined in this study, we included the Mini-K scale (Figueredo et al., 2006), which was designed to measure individual differences in fast vs. slow life history strategies. We decided to expand the measures of ‘impulsivity’ because human life history research encompasses a wide range of traits as dependent variables, despite a lack of evidence that those traits do in fact cluster together (Sear, 2020). For example, risky behaviours may refer to at least two distinct constructs: activities that would increase the likelihood of undesirable outcomes (e.g. unprotected sex, drug use) and willingness to accept outcome variance, the latter being standard operationalisation in laboratory studies (Pepper & Nettle, 2017). Therefore, we considered it better to expand our independent variables to cover as many purported life history traits as possible. Although some scholars question its psychometric properties (e.g. Richardson et al., 2017), the Mini-K scale is often used to measure position on the fast–slow continuum (e.g. Figueredo et al., 2014). Accordingly, we included it as an additional measure.

We preregistered the protocol for this study on the Open Science Framework (OSF; https://osf.io/tuav4/). This study was approved by the institutional review board at the first author's institute.

## Method

### Participants

A sample of 4,579 Japanese community-based participants was recruited in September 2020 through an online survey service (Cross Marketing Inc., Japan). Given that our primary dependent variable was number of children, we specifically recruited participants aged 40–45 years, surpassing the Japanese average first-marriage age of 30.7 and 29.0 years for men and women, respectively (Ministry of Health, Labour and Welfare, 2016). As preregistered, we included a stringent attention check (i.e. asking participants to ignore an entire page of the survey; Miura & Kobayashi, 2015) along with two standard attention checks (i.e. asking participants to choose a specific option). Consequently, a large number of participants (3,446 participants) failed. We also excluded 441 participants, most of whom were not in the required age range. We retained 692 participants (352 women, 337 men and 3 not reported; 42.72 ± 1.73 years old), which was slightly below the preregistered sample size of 700, for subsequent analyses.

### Independent variables

The childhood SES measure consisted of three items (e.g. ‘My family usually had enough money for things when I was growing up’), rated on a nine-point scale (1 = strongly disagree to 9 = strongly agree; Cronbach's *α* = 0.79). The temporal discounting task consisted of 20 choices between an immediate small reward (e.g. receiving JPY 4100 tomorrow) and a larger delayed reward (e.g. receiving JPY 5,100 after 33 days). The risk-taking task consisted of 20 choices between definitely earning a fixed amount of money and earning a larger amount of money with a certain probability (e.g. earning JPY 5,600 for sure vs. a 54% chance of earning JPY 6500). The frequency of choosing the immediate reward options and gamble options was used as the index of impulsivity for the temporal discounting task and risk taking task, respectively. These materials, translated into Japanese by the authors, were adapted from Griskevicius et al. (2013).

In addition, participants filled out the Japanese version 20-item Mini-K scale (Figueredo et al., 2006; Kawamoto, 2015), designed to measure individual differences in fast vs. slow life history strategies (e.g. ‘I often make plans in advance’). Participants rated each item on a seven-point scale (−3 = strongly disagree to 3 = strongly agree). We excluded two items that assess participants’ childhood experiences, which have some overlap with the independent variable (i.e. childhood SES). The Mini-K score (Cronbach's *α* = 0.84) represents orientation towards the slow life history strategy; higher scores indicate lower impulsivity.

### Dependent variables

The dependent variables were proxy indices of participants’ fitness (i.e. number of children, marriage experience, annual household income, subjective SES, life satisfaction). A straightforward fitness index was reproductive success, operationalised as the *number of children* that participants had throughout their lives. We explicitly asked them to include any children from previous marriages. *Marriage experience* (i.e. lifelong singlehood vs. having at least one marriage) was also included; lifelong singles are considered less reproductively successful. *Annual household income* and *subjective SES* were included because wealth enables greater investment in offspring. Participants reported their household income last year, broken down into six ranges: <1.5, [1.5, 3), [3, 5), [5, 7), [7, 10) and ≥10 (units of million JPY). Subjective SES was measured using the MacArthur Scale (Adler et al., 2000), requiring participants to choose one of 10 SES-level rungs on a ladder. *Life satisfaction* was included, assuming that their assessment of their own quality of life may be associated with some aspects of biological fitness. It was measured using the Satisfaction With Life Scale (Diener et al., 1985) consisting of five items (e.g. ‘In most ways my life is close to my ideal’), rated on a seven-point scale (1 = strongly disagree to 7 = strongly agree; Cronbach's *α* = 0.90).

### Analyses

We divided the participants into four groups by bivariate median splits, combining childhood SES with one of the three impulsivity indices (i.e. temporal discounting, risk taking, or life history strategy). The four groups were: low impulsivity, high childhood SES (group 1, fitness denoted as *β*_0_ in Table 1); high impulsivity, high childhood SES (group 2, *α*_0_); low impulsivity, low childhood SES (group 3, *β*_1_); and high impulsivity, low childhood SES (group 4, *α*_1_). Based on the hypothesised inequality, we ran a set of three planned contrasts with weights of (1, −1, 0, 0), (0, 0, 1, −1) and (0.5, 0.5, −0.5, −0.5) for (group 1, group 2, group 3, group 4). The first two contrasts were designed to test *β*_0_ > *α*_0_ and *α*_1_ > *β*_1_, respectively. The third contrast corresponded to the comparison between high and low childhood SES groups, included to test the basic presumption that childhood economic harshness adversely influences later fitness. The data used in the reported analyses and the R code are available from OSF (https://osf.io/tuav4/).

## Results

Tables 2 and 3 show descriptive statistics and correlation matrices of the variables of interest (Table 2 for the independent variables and Table 3 for the dependent variables). We first examined whether childhood SES was negatively associated with impulsivity. Contrary to previous studies, childhood SES was not significantly associated with temporal discounting (*r*_668_ = −0.06) or risk taking (*r*_673_ = 0.01). However, it was significantly associated with a self-rated slow life history strategy (i.e. the Mini-K score; *r*_663_ = 0.26, *p* < 0.001).

**Table 2.** Descriptive statistics and correlation matrix of the independent variables

Variable	*N*	Mean	Median	SD	1	2	3
1. Temporal discounting	672	5.16	4.00	5.34	−		
2. Risk taking	676	6.61	4.00	6.74	0.05	−	
3. Mini-K	666	0.35	0.38	0.84	−0.02	0.04	−
4. Childhood SES	687	4.61	4.67	1.57	−0.06	0.01	0.26***

*** *p* < 0.001.

**Table 3.** Descriptive statistics and correlation matrix of the dependent variables

Variable	*N*	Mean	SD	1	2	3	4
1. Number of children	686	0.69	0.99	−			
2. Marriage experience	687	0.57	−	0.59***	−		
3. Annual household income	682	3.49	1.46	0.25***	0.34***	−	
4. Subjective SES	688	4.63	1.83	0.26***	0.37***	0.52***	−
5. Life satisfaction	681	3.38	1.29	0.28***	0.39***	0.37***	0.55***

*Note*: Marriage experience – 0 = lifetime singlehood, 1 = having at least one marriage.

*** *p* < 0.001.

**Figure 1.**
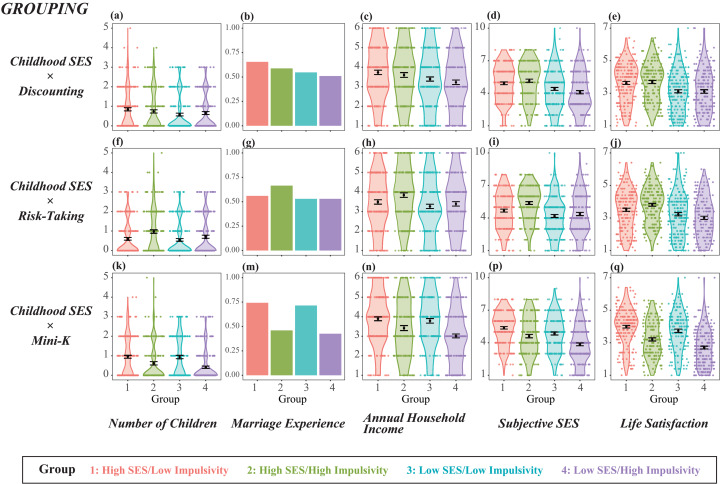
Distribution of fitness indices of four groups divided by medians of childhood socioeconomic status (SES) and impulsivity. Note: the hypothesis predicts a horizontally mirrored J-shape – highest, second highest, lowest and second lowest for groups 1–4, respectively. Results based on childhood SES × temporal discounting grouping, childhood SES × risk-taking grouping, and childhood SES × Mini-K grouping are presented as (a–e), (f–j) and (k–q), respectively. The dependent variables were number of children for (a), (f) and (k), marriage experience for (b), (g) and (m); annual household income for (c), (h) and (n); subjective SES for (d), (i) and (p); and life satisfaction for (e), (j) and (q).

Figure 1 shows the distributions of the five fitness indices of groups 1–4. The four groups were divided by childhood SES and impulsivity, which are operationalised as temporal discounting in Figure 1a–e, risk-taking tendency in Figure 1f–j and Mini-K score in Figure 1k–q. Although the hypothesised inequality predicts a horizontally mirrored J-shaped pattern, none of the figures shows this (except Figure 1a, which exhibits a slight tendency towards a horizontally mirrored J-shape).

When impulsivity was operationalised by temporal discounting (Figures 1a–e), contrasts 1 and 2 yielded no significant results for any of the five fitness indices (see also Table S1). When impulsivity was operationalised by risk-taking tendency (Figures 1f–j), although contrast 2 revealed no significant results, contrast 1 showed significant effects opposite to the hypothesis: group 2 reported having more children (*b* = −0.24, SE = 0.06, *p* < 0.001), higher annual household income (*b* = −0.17, SE = 0.08, *p* < 0.05), higher subjective SES (*b* = −0.35, SE = 0.10, *p* < 0.001) and higher life satisfaction (*b* = −0.15, SE = 0.07, *p* < 0.05) than group 1 (see Table S1 for details).

When impulsivity was operationalised by Mini-K score (Figures 1k–q, see also Table S1), regardless of childhood SES (i.e. in both contrasts 1 and 2), lower impulsivity was consistently associated with higher fitness: more children (*b* = 0.22, SE = 0.07, *p* < 0.001 and *b* = 0.41, SE = 0.07, *p* < 0.001 for contrasts 1 and 2, respectively), higher likelihood of marriage (*b* = 0.61, SE = 0.12, *p* < 0.001 and *b* = 0.61, SE = 0.12, *p* < 0.001), higher annual household income (*b* = 0.24, SE = 0.08, *p* < 0.01 and *b* = 0.39, SE = 0.08, *p* < 0.001), higher subjective SES (*b* = 0.38, SE = 0.10, *p* < 0.001 and *b* = 0.50, SE = 0.09, *p* < 0.001) and higher life satisfaction (*b* = 0.39, SE = 0.07, *p <* 0.001 and *b* = 0.52, SE = 0.06, *p* < 0.001). This pattern predicts that impulsivity was selected against regardless of childhood economic harshness.

Contrast 3 (high vs. low childhood SES) was tested three times, although redundant, for the three grouping schemes. Therefore, as shown in Table S1, despite grouping schemes, groups 1 and 2 (high childhood SES groups) were associated with higher fitness than groups 3 and 4 (low childhood SES groups) on every measure, confirming the basic presumption that low childhood SES adversely influenced later fitness. Parenthetically, this result suggests the criterion validity of our childhood SES measure as well as fitness indices: despite contemporary Japan's low fertility rate (1.36 in 2019) and ever increasing lifetime unmarried rate (23.4 and 14.1 for males and females, respectively, in 2015; Ministry of Health, Labour, and Welfare, 2020), the influence of the theoretically relevant variable (i.e. childhood SES) was observed for the number of children and marriage experience.

We conducted four sets of robustness checks. First, we redefined the four groups using the upper and lower tertiles of childhood SES and impulsivity scores (Table S2, Figure S1). Second, we focused on participants experiencing real economic threats whose annual household income decreased from the previous year, possibly owing to COVID-19 (Table S3, Figure S2). Third, the same analyses were conducted separately by sex (Tables S4 and S5, Figures S3 and S4). Fourth, we tested the hypothesis without splitting the participants into four groups. In a series of regression analyses that treated childhood SES and impulsivity measures as continuous variables, each of the five fitness measures was regressed on childhood SES, one of the three impulsivity measures (either temporal discounting, risk-taking tendency or Mini-K score) and their interaction term (Table S6 for the entire sample, Table S7 for the subsample of participants whose annual income decreased from the previous year, Table S8 for the male sample and Table S9 for the female sample). These robustness checks revealed a mostly identical pattern. In addition, since the fast strategy may result in a greater likelihood of divorce and remarriage, we conducted the three planned contrasts using the number of marriages as the dependent variable. The results were mostly similar to the planned contrasts that included the other fitness indices as the dependent variables (see Table S10).

## Discussion

We examined the evolvability of the impulsivity reaction norm. Although the results confirmed the basic presumption that childhood economic harshness adversely influenced participants’ later fitness, none of the other results supported the impulsivity reaction norm's evolvability: when operationalised as risk-taking tendency, impulsivity was associated with higher fitness among individuals with high, but not low, childhood SES (this pattern, however, was not replicated in our subsequent unpublished study including only male participants). When operationalised by Mini-K score, it was associated with lower fitness regardless of childhood SES.

The present study failed to replicate the results of previous studies (Griskevicius et al., 2013). In particular, childhood SES was not significantly associated with either risk taking or temporal discounting. Given the robust association between childhood SES and BCD (Pepper & Nettle, 2017), the present study's operationalisation of impulsivity might have provided inadequate indices of impulsivity. For example, one could argue that the temporal discounting and risk-taking tasks should have been incentivised (but see Amir et al., 2018, which reported no systematic differences between incentivised and non-incentivised risk-taking tasks). Mishra et al. (2017) recently proposed a model of risk-taking (relative state model) that distinguishes two types of risk-taking behaviours, need-based and ability-based risk-taking; the former is motivated by poor environments, while the latter is motivated by superior abilities (i.e. the prospect of successful risk-taking). In future studies, it is worthwhile not only to incentivise risk-taking tasks but also to distinguish subtypes of risk-taking and impulsivity based on such a nuanced model.

One limitation is that we assessed fitness in a modern, industrialised society that is largely different from the environment of evolutionary adaptedness (EEA). For example, if low SES conditions in contemporary Japan are still more benign compared with harsh conditions in EEA, the present study may not be a fair test of the hypothesised phenotypic plasticity. Nonetheless, it is worth noting that childhood SES was in fact positively associated with every measure of fitness in this study. Moreover, careful analyses revealed some comparability of the modern and ancestral environments (i.e. positive association between wealth and the number of children in both modern and ancestral environments; Nettle & Pollet, 2008). Nevertheless, this particular result does not necessarily imply that the comparability between the modern and ancestral environments extends to other aspects. For example, one could argue that impulsivity is an effective strategy for disadvantaged individuals only in EEA but not in the modern environment. Since there is a wide range of differences between the modern environment and EEA, or the so-called evolutionary mismatch problem (Li et al., 2018), it is informative to replicate this study in populations that maintain traditional lifestyles.

We admit that this study does not disprove the evolvability of human reaction norms as a whole. This study only tested the evolvability of impulsivity in response to childhood economic harshness. There are other independent and dependent variables that have attracted researchers’ attention in the context of life history theory in psychology. For example, timing of puberty and parental strategies are oft-studied life history traits (i.e. dependent variables), and childhood mortality/morbidity and unpredictability are oft-studied environmental (independent) variables (Ellis et al., 2009). Therefore, future studies need to include a wider range of measures of childhood environments and life history traits in order to fully test the evolvability of any form of phenotypic plasticity in response to early environments.

In summary, this study does not reveal any evidence of the evolvability of the impulsivity reaction norm in response to childhood economic harshness. Therefore, we urge researchers to critically assess the impulsivity reaction norm, especially whether the adaptationist explanation is better supported by empirical data than by-product explanations.

## Data Availability

The materials, protocol, data and analytical codes that support the findings of this study are openly available from the Open Science Framework at https://osf.io/tuav4/.

## Supplementary material

For supplementary material accompanying this paper visit https://doi.org/10.1017/ehs.2022.22. click here to view supplementary material
