# Supplementary material for: Can impulsivity evolve in response to childhood environmental harshness?
Source: Evol Hum Sci. 2022 May 24;4:e21. doi: 10.1017/ehs.2022.22 (PMC10426006; doi:10.1017/ehs.2022.22)
Supplement: Supplementary file 1 [file S2513843X22000226sup001.pdf]

<u>Supplementary Materials</u>

Can Impulsivity Evolve in Response to Childhood Environmental Harshness?

Atsushi Kometani (Kobe University) Yohsuke Ohtsubo (University of Tokyo)

Table S1

Results of a Series of Planned Contrast Analyses

	Variable Used for Grouping								
	Temporal Discounting			Risk Taking			Mini K		
	*n*s = 173, 149, 190, and 158 for Groups 1, 2, 3, and 4, respectively			*n*s = 167, 160, 183, and 165 for Groups 1, 2, 3, and 4, respectively			*n*s = 185, 138, 145, and 197 for Groups 1, 2, 3, and 4, respectively		
	Corresponding to Figs. 1a to 1e			*Corresponding to Figs. 1f to 1j*			*Corresponding to Figs. 1k to 1q*		
	b	*se*	*p*	*b*	*se*	*p*	*b*	*se*	*p*
Number of Children									
Contrast 1	0.07	0.06	.271	***−0.24***	0.06	<.001	**0.22**	0.07	<.001
Contrast 2	−0.06	0.07	.373	−0.13	0.07	.059	***0.41***	0.07	<.001
Contrast 3	**0.25**	0.09	.007	**0.21**	0.09	.023	**0.20**	0.10	.038
Marriage Experience (0 = lifetime singlehood, 1 = having at least one marriage)									
Contrast 1	0.14	0.12	.218	−0.22	0.12	.055	**0.61**	0.12	<.001
Contrast 2	0.08	0.11	.482	−0.00	0.11	.994	***0.61***	0.12	<.001
Contrast 3	**0.38**	0.16	.015	**0.34**	0.16	.030	0.14	0.17	.410
Annual Household Income									
Contrast 1	0.07	0.08	.420	***−0.17***	0.08	.032	**0.24**	0.08	.003
Contrast 2	0.08	0.08	.297	−0.06	0.08	.424	***0.39***	0.08	<.001
Contrast 3	**0.36**	0.11	.002	**0.34**	0.11	.003	**0.26**	0.11	.022
Subjective SES									
Contrast 1	−0.12	0.10	.241	***−0.35***	0.10	<.001	**0.38**	0.10	<.001
Contrast 2	0.15	0.10	.114	−0.09	0.10	0.34	***0.50***	0.09	<.001
Contrast 3	**0.79**	0.14	<.001	**0.75**	0.14	<.001	**0.64**	0.14	<.001
Life Satisfaction									
Contrast 1	−0.03	0.07	.678	***−0.15***	0.07	.031	**0.39**	0.07	<.001
Contrast 2	0.01	0.07	.932	0.11	0.07	.108	***0.52***	0.06	<.001
Contrast 3	**0.55**	0.10	<.001	**0.54**	0.10	<.001	**0.37**	0.09	<.001

Note. Number of children was submitted to Poisson regression analyses; marriage experience was submitted to logistic regression analyses. **Bold** coefficients indicate significant difference in the hypothesised direction. ***Bold italic*** coefficients indicate significant difference opposite to the hypothesised direction.

Figure S1

Distribution of fitness indices of four groups divided into the upper/lower tertiles of childhood SES and impulsivity.

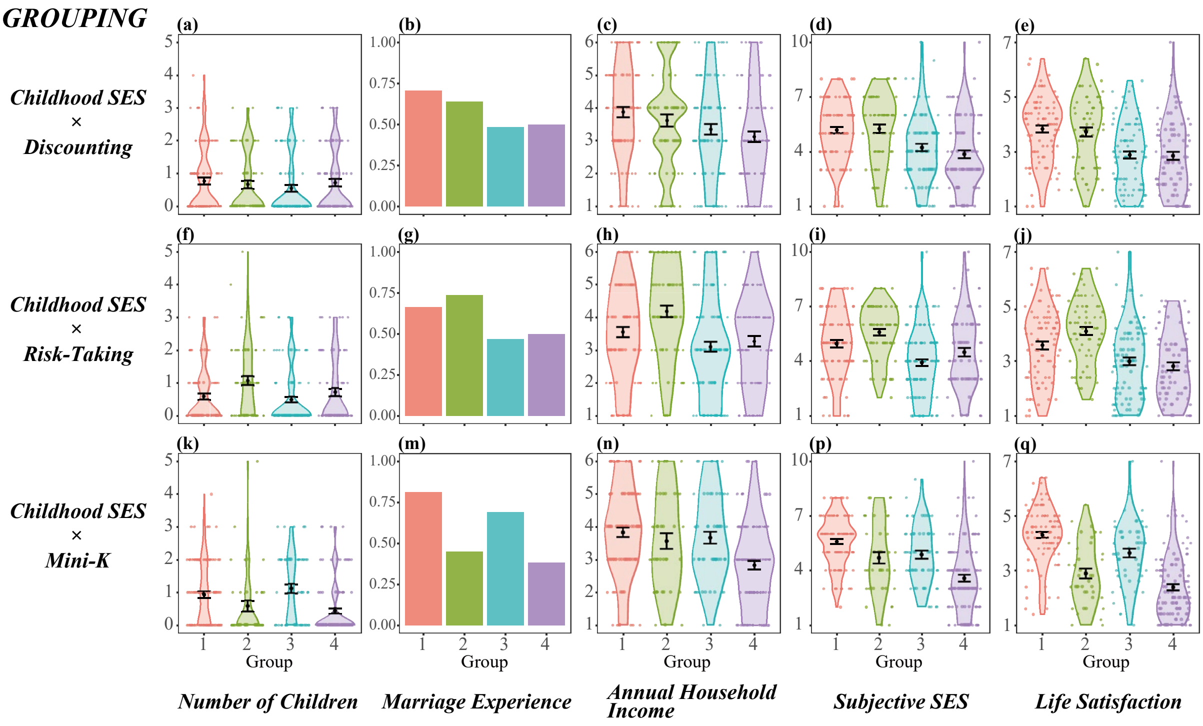

Note. The hypothesised inequality predicts a horizontally mirrored J-shape: highest, second-highest, lowest, and second-lowest for Groups 1, 2, 3, and 4, respectively. Results based on childhood SES × temporal discounting grouping are presented as Figures S1a to S1e. Results based on childhood SES × risk-taking grouping are presented as Figures S1f to S1j. Results based on childhood SES × Mini-K grouping are presented as Figures S1k to S1q. The dependent variables were the number of children for Figures S1a, S1f, and S1k, marriage experience for Figures S1b, S1g, and S1m, annual household income for Figures S1c, S1h, and S1n, subjective SES for Figures S1d, S1i, and S1p, and life satisfaction for Figures S1e, S1j and S1q.

Table S2

Results of a Series of Planned Contrast Analyses for Four Groups Divided into Upper/Lower Tertiles of the Childhood SES and Impulsivity

	Variable Used for Grouping								
	Temporal Discounting			Risk Taking			Mini K		
	*n*s = 86, 62, 85, and 82 for Groups 1, 2, 3, and 4, respectively			*n*s = 84, 66, 98, and 73 for Groups 1, 2, 3, and 4, respectively			*n*s = 93, 40, 55, and 102 for Groups 1, 2, 3, and 4, respectively		
	Corresponding to Figs. S1a to S1e			*Corresponding to Figs. S1f to S1j*			*Corresponding to Figs. S1k to S1q*		
	b	*se*	*p*	*b*	*se*	*p*	*b*	*se*	*p*
Number of Children									
Contrast 1	0.08	0.10	.410	***−0.30***	0.09	.001	**0.24**	0.12	.039
Contrast 2	−0.14	0.10	.165	−0.20	0.10	.054	***0.47***	0.10	<.001
Contrast 3	0.13	0.14	.368	**0.30**	0.14	.032	0.06	0.15	.711
Marriage Experience (0 = lifetime singlehood, 1 = having at least one marriage)									
Contrast 1	0.15	0.18	.397	−0.18	0.18	.321	**0.84**	0.21	<.001
Contrast 2	−0.04	0.16	.820	−0.06	0.16	.693	***0.64***	0.18	<.001
Contrast 3	**0.76**	0.24	.001	**0.92**	0.24	<.001	0.47	0.27	.084
Annual Household Income									
Contrast 1	0.13	0.12	.288	***−0.31***	0.12	.009	0.13	0.13	.303
Contrast 2	0.11	0.11	.334	−0.09	0.11	.414	***0.41***	0.12	<.001
Contrast 3	**0.51**	0.17	.002	**0.67**	0.16	<.001	**0.44**	0.17	.011
Subjective SES									
Contrast 1	−0.04	0.15	.819	***−0.32***	0.15	.034	**0.46**	0.16	.005
Contrast 2	0.19	0.14	.189	−0.27	0.14	.052	***0.65***	0.14	<.001
Contrast 3	**1.21**	0.21	<.001	**1.08**	0.21	<.001	**0.93**	0.22	<.001
Life Satisfaction									
Contrast 1	0.05	0.11	.621	***−0.26***	0.11	.017	**0.71**	0.11	<.001
Contrast 2	0.02	0.10	.842	0.09	0.10	.383	***0.63***	0.10	<.001
Contrast 3	**0.92**	0.15	<.001	**0.96**	0.15	<.001	**0.59**	0.14	<.001

Note. Number of children was submitted to Poisson regression analyses; marriage experience was submitted to logistic regression analyses. **Bold** coefficients indicate significant difference in the hypothesised direction. ***Bold italic*** coefficients indicate significant difference opposite to the hypothesised direction.

Figure S2

Distribution of fitness indices of four groups median split into by childhood SES and impulsivity. Only individuals whose annual household income decreased from the last year were included.

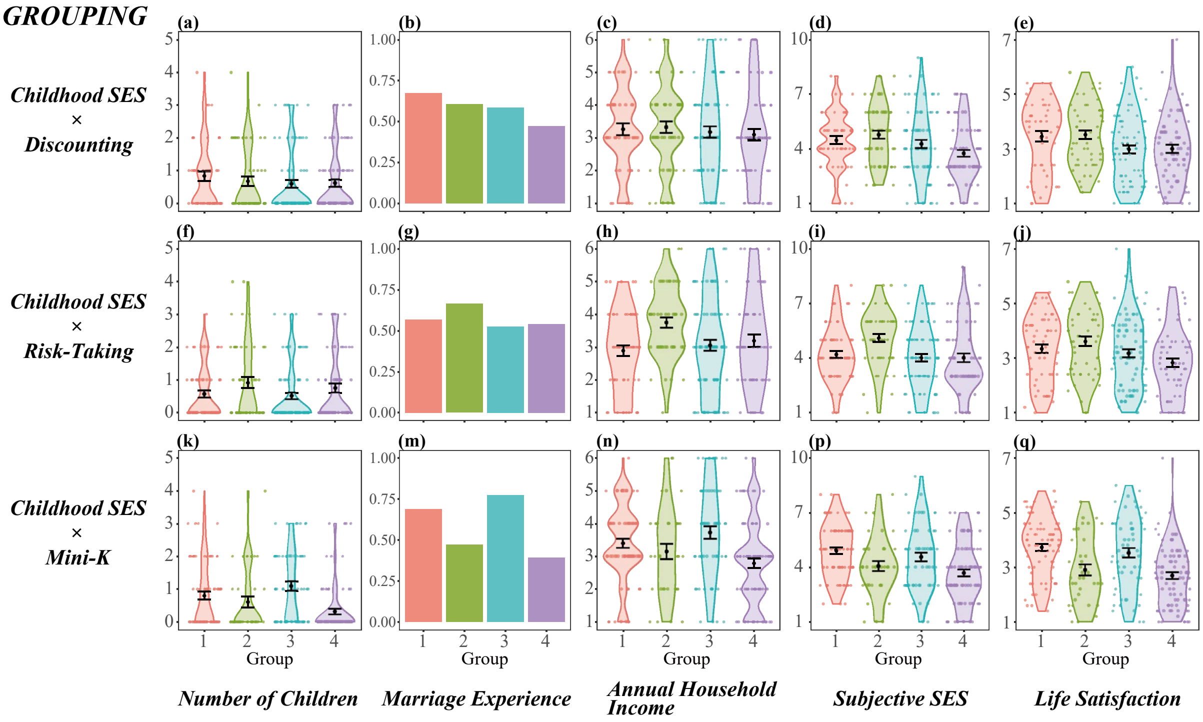

Note. The hypothesised inequality predicts a horizontally mirrored J-shape: highest, second-highest, lowest, and second-lowest for Groups 1, 2, 3, and 4, respectively. Results based on childhood SES × temporal discounting grouping are presented as Figures S2a to S2e. Results based on childhood SES × risk-taking grouping are presented as Figures S2f to S2j. Results based on childhood SES × Mini-K grouping are presented as Figures S2k to S2q. The dependent variables were the number of children for Figures S2a, S2f, and S2k, marriage experience for Figures S2b, S2g, and S2m, annual household income for Figures S2c, S2h, and S2n, subjective SES for Figures S2d, S2i, and S2p, and life satisfaction for Figures S2e, S2j and S2q.

Table S3

Results of a Series of Planned Contrast Analyses (Only Participants Whose Annual Household Income Decreased from the Last Year)

	Variable Used for Grouping								
	Temporal Discounting			Risk Taking			Mini K		
	*n*s = 49, 54, 72, and 70 for Groups 1, 2, 3, and 4, respectively			*n*s = 59, 48, 80, and 61 for Groups 1, 2, 3, and 4, respectively			*n*s = 72, 36, 58, and 84 for Groups 1, 2, 3, and 4, respectively		
	Corresponding to Figs. S2a to S2e			*Corresponding to Figs. S2f to S2j*			*Corresponding to Figs. S2k to S2q*		
	b	*se*	*p*	*b*	*se*	*p*	*b*	*se*	*p*
Number of Children									
Contrast 1	0.10	0.11	.361	***−0.24***	0.12	.038	0.14	0.13	.277
Contrast 2	−0.02	0.11	.862	−0.19	0.11	.078	***0.63***	0.12	<.001
Contrast 3	0.22	0.16	.156	0.15	0.16	.334	0.19	0.17	.272
Marriage Experience (0 = lifetime singlehood, 1 = having at least one marriage)									
Contrast 1	0.15	0.21	.465	−0.21	0.20	.305	**0.46**	0.21	.030
Contrast 2	0.23	0.17	.183	−0.03	0.17	.851	***0.84***	0.19	<.001
Contrast 3	0.46	0.27	.084	0.35	0.27	.182	−0.06	0.29	.838
Annual Household Income									
Contrast 1	−0.03	0.14	.829	***−0.43***	0.13	.001	0.13	0.14	.358
Contrast 2	0.03	0.12	.779	−0.07	0.11	.522	***0.46***	0.11	<.001
Contrast 3	0.15	0.18	.402	0.20	0.17	.253	0.01	0.18	.960
Subjective SES									
Contrast 1	−0.15	0.17	.383	***−0.45***	0.16	.006	**0.43**	0.17	.011
Contrast 2	0.26	0.14	.065	−0.00	0.14	.990	***0.44***	0.14	.002
Contrast 3	**0.62**	0.22	.005	**0.64**	0.22	.003	0.37	0.22	.089
Life Satisfaction									
Contrast 1	−0.03	0.12	.813	−0.14	0.12	.265	**0.42**	0.12	<.001
Contrast 2	−0.01	0.10	.948	0.17	0.11	.113	***0.42***	0.10	<.001
Contrast 3	**0.50**	0.16	.002	**0.48**	0.16	.003	0.21	0.16	.179

Note. Number of children was submitted to Poisson regression analyses; marriage experience was submitted to logistic regression analyses. **Bold** coefficients indicate significant difference in the hypothesised direction. ***Bold italic*** coefficients indicate significant difference opposite to the hypothesised direction.

Figure S3

Distribution of fitness indices of four groups median split into by childhood SES and impulsivity (Male Participants).

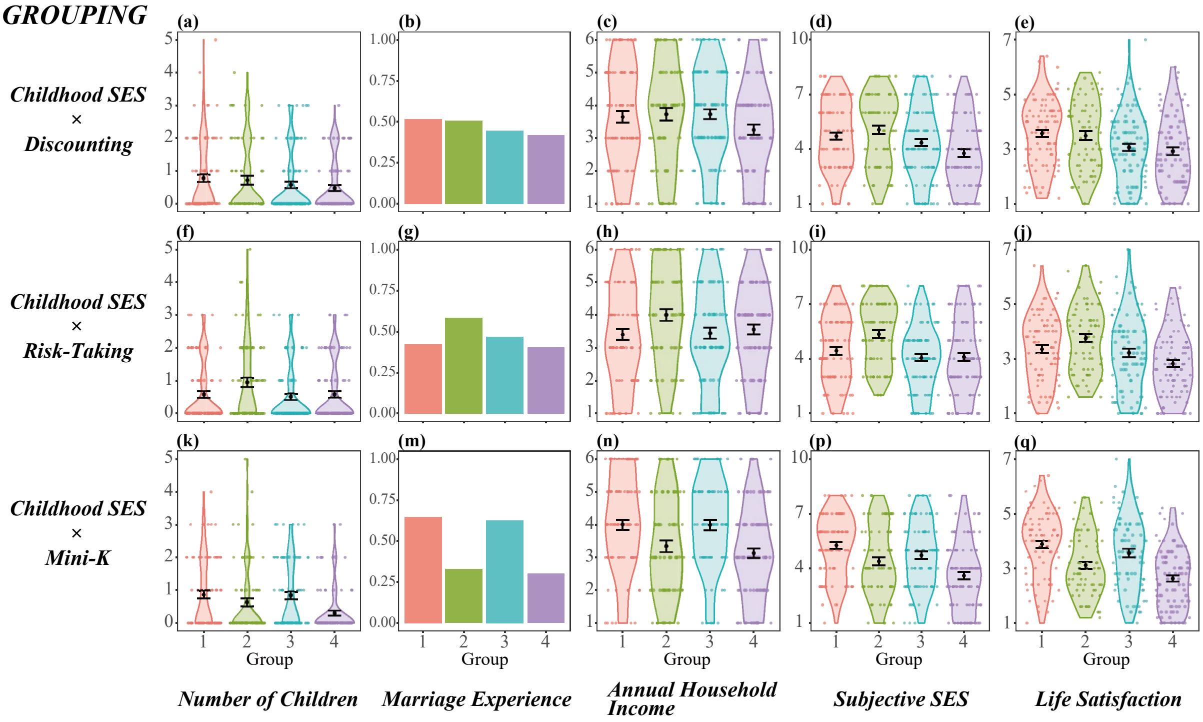

Note. The hypothesised inequality predicts a horizontally mirrored J-shape: highest, second-highest, lowest, and second-lowest for Groups 1, 2, 3, and 4, respectively. Results based on childhood SES × temporal discounting grouping are presented as Figures S3a to S3e. Results based on childhood SES × risk-taking grouping are presented as Figures S3f to S3j. Results based on childhood SES × Mini-K grouping are presented as Figures S3k to S3q. The dependent variables were the number of children for Figures S3a, S3f, and S3k, marriage experience for Figures S3b, S3g, and S3m, annual household income for Figures S3c, S3h, and S3n, subjective SES for Figures S3d, S3i, and S3p, and life satisfaction for Figures S3e, S3j and S3q.

Table S4

Results of a Series of Planned Contrast Analyses (Male Participants)

	Variable Used for Grouping								
	Temporal Discounting			Risk Taking			Mini K		
	*n*s = 88, 65, 94, and 80 for Groups 1, 2, 3, and 4, respectively			*n*s = 83, 73, 81, and 90 for Groups 1, 2, 3, and 4, respectively			*n*s = 85, 71, 76, and 93for Groups 1, 2, 3, and 4, respectively		
	Corresponding to Figs. S3a to S3e			*Corresponding to Figs. S3f to S3j*			*Corresponding to Figs. S3k to S3q*		
	b	*se*	*p*	*b*	*se*	*p*	*b*	*se*	*p*
Number of Children									
Contrast 1	0.04	0.10	.695	***−0.25***	0.10	.008	0.16	0.10	.090
Contrast 2	0.10	0.11	.369	−0.07	0.10	.526	***0.51***	0.11	<.001
Contrast 3	**0.36**	0.14	.011	**0.31**	0.14	.029	**0.38**	0.15	.010
Marriage Experience (0 = lifetime singlehood, 1 = having at least one marriage)									
Contrast 1	0.02	0.16	.907	***−0.33***	0.16	.046	**0.66**	0.17	<.001
Contrast 2	0.06	0.15	.701	0.13	0.16	.396	***0.68***	0.16	<.001
Contrast 3	0.32	0.23	.152	0.27	0.23	.238	0.11	0.24	.649
Annual Household Income									
Contrast 1	−0.04	0.12	.769	***−0.30***	0.12	.013	**0.33**	0.12	.005
Contrast 2	***0.23***	0.11	.042	−0.06	0.11	.587	***0.43***	0.11	<.001
Contrast 3	0.21	0.17	.218	0.21	0.17	.209	0.11	0.16	.477
Subjective SES									
Contrast 1	−0.17	0.16	.263	***−0.46***	0.15	.003	**0.44**	0.15	.003
Contrast 2	***0.29***	0.15	.046	−0.03	0.14	.862	***0.56***	0.14	<.001
Contrast 3	**0.82**	0.21	<.001	**0.82**	0.21	<.001	**0.66**	0.21	.001
Life Satisfaction									
Contrast 1	0.04	0.10	.711	−0.20	0.10	.050	**0.38**	0.10	<.001
Contrast 2	0.07	0.10	.457	***0.20***	0.10	.042	***0.46***	0.09	<.001
Contrast 3	**0.55**	0.14	<.001	**0.53**	0.14	<.001	**0.42**	0.13	.002

Note. Number of children was submitted to Poisson regression analyses; marriage experience was submitted to logistic regression analyses. **Bold** coefficients indicate significant difference in the hypothesised direction. ***Bold italic*** coefficients indicate significant difference opposite to the hypothesised direction.

Figure S4

Distribution of fitness indices of four groups median split into by childhood SES and impulsivity (Female Participants).

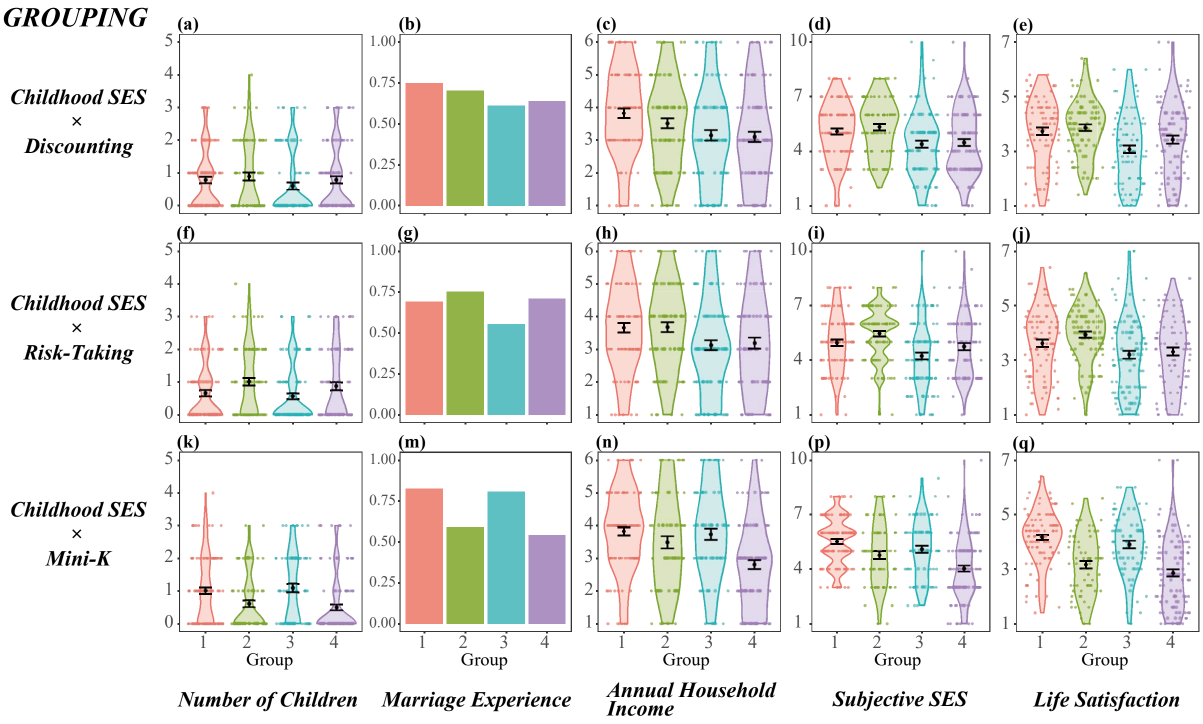

Note. The hypothesised inequality predicts a horizontally mirrored J-shape: highest, second-highest, lowest, and second-lowest for Groups 1, 2, 3, and 4, respectively. Results based on childhood SES × temporal discounting grouping are presented as Figures S4a to S4e. Results based on childhood SES × risk-taking grouping are presented as Figures S4f to S4j. Results based on childhood SES × Mini-K grouping are presented as Figures S4k to S4q. The dependent variables were the number of children for Figures S4a, S4f, and S4k, marriage experience for Figures S4b, S4g, and S4m, annual household income for Figures S4c, S4h, and S4n, subjective SES for Figures S4d, S4i, and S4p, and life satisfaction for Figures S4e, S4j and S4q.

Table S5

Results of a Series of Planned Contrast Analyses (Female Participants)

	Variable Used for Grouping								
	Temporal Discounting			Risk Taking			Mini K		
	ns = 89, 79, 83, and 89 for Groups 1, 2, 3, and 4, respectively			*ns* = 88, 82, 96, and 79 for Groups 1, 2, 3, and 4, respectively			*ns* = 101, 66, 68, and 103 for Groups 1, 2, 3, and 4, respectively		
	Corresponding to Figs. S4a to S4e			*Corresponding to Figs. S4f to S4j*			*Corresponding to Figs. S4k to S4q*		
	b	*se*	*p*	*b*	*se*	*p*	*b*	*se*	*p*
Number of Children									
Contrast 1	−0.07	0.09	.426	***−0.22***	0.09	.012	**0.26**	0.09	.006
Contrast 2	−0.14	0.09	.140	**−0.22**	0.09	.016	***0.39***	0.09	<.001
Contrast 3	0.20	0.13	.109	0.15	0.13	.229	0.06	0.13	.629
Marriage Experience (0 = lifetime singlehood, 1 = having at least one marriage)									
Contrast 1	0.11	0.18	.517	−0.16	0.17	.361	**0.60**	0.18	<.001
Contrast 2	−0.06	0.16	.725	**−0.34**	0.16	.034	***0.63***	0.18	<.001
Contrast 3	**0.46**	0.24	.049	0.41	0.24	.085	0.16	0.26	.532
Annual Household Income									
Contrast 1	0.16	0.11	.153	−0.00	0.11	.986	0.17	0.11	.114
Contrast 2	0.02	0.11	.833	−0.03	0.11	.773	***0.46***	0.11	<.001
Contrast 3	**0.55**	0.15	<.001	**0.52**	0.15	<.001	**0.39**	0.15	.011
Subjective SES									
Contrast 1	−0.12	0.13	.350	***−0.26***	0.13	.044	**0.38**	0.13	.003
Contrast 2	−0.06	0.13	.666	**−0.26**	0.13	.036	***0.53***	0.12	<.001
Contrast 3	**0.80**	0.18	<.001	**0.73**	0.18	<.001	**0.61**	0.18	<.001
Life Satisfaction									
Contrast 1	−0.06	0.10	.541	−0.15	0.10	.114	**0.50**	0.09	<.001
Contrast 2	−0.18	0.10	.066	−0.06	0.10	.515	***0.52***	0.09	<.001
Contrast 3	**0.55**	0.14	<.001	**0.53**	0.14	<.001	**0.29**	0.13	.023

Note. Number of children was submitted to Poisson regression analyses; marriage experience was submitted to logistic regression analyses. **Bold** coefficients indicate significant difference in the hypothesised direction. ***Bold italic*** coefficients indicate significant difference opposite to the hypothesised direction.

Table S6

Results of a Series of Multiple Regression Analyses

	Impulsivity Variable								
	Temporal Discounting			Risk Taking			Mini K		
	b	*se*	*p*	*b*	*se*	*p*	*b*	*se*	*p*
Number of Children									
Childhood SES	.120	.047	.011	.086	.047	.068	.016	.051	.752
(Impulsivity)	.035	.046	.444	.173	.045	<.001	.406	.049	<.001
Interaction	−.046	.044	.299	.079	.044	.072	−.046	.045	.313
Marriage Experience (0 = lifetime singlehood, 1 = having at least one marriage)									
Childhood SES	.276	.081	<.001	.261	.080	.001	.063	.090	.485
(Impulsivity)	−.090	.079	.259	.123	.080	.124	.898	.103	<.001
Interaction	−.062	.076	.414	.094	.079	.232	.099	.092	.284
Annual Household Income									
Childhood SES	.162	.038	<.001	.167	.038	<.001	.110	.039	.005
(Impulsivity)	−.081	.038	.036	.075	.038	.049	.200	.039	<.001
Interaction	−.007	.036	.839	.061	.037	.102	−.025	.033	.449
Subjective SES									
Childhood SES	.261	.038	<.001	.264	.037	<.001	.177	.037	<.001
(Impulsivity)	−.023	.037	.539	.143	.037	<.001	.328	.037	<.001
Interaction	.037	.035	.291	.018	.036	.623	−.033	.032	.293
Life Satisfaction									
Childhood SES	.273	.038	<.001	.274	.037	<.001	.130	.035	<.001
(Impulsivity)	−.006	.038	.866	.027	.037	.467	.480	.035	<.001
Interaction	−.019	.036	.594	.103	.036	.005	−.000	.029	.996

Note. Childhood SES and impulsivity were standardised for the set of these multiple regression analyses. Number of children was submitted to Poisson regression analyses, and marriage experience was submitted to logistic regression analyses. For the remaining dependent variables (i.e., annual household income, subjective SES, and life satisfaction), reported regression coefficients are standardized coefficients. For the two shaded cells (one significant and one marginally significant interaction effects), we conducted a series of simple slope tests. Neither of the interactions was consistent with the hypothesis (the slope is positive for low childhood SES group and negative for high childhood SES group).

Table S7

Results of a Series of Multiple Regression Analyses (Only Participants Whose Annual Household Income Decreased from the Last Year)

	Impulsivity Variable								
	Temporal Discounting			Risk Taking			Mini K		
	b	*se*	*p*	*b*	*se*	*p*	*b*	*se*	*p*
Number of Children									
Childhood SES	.141	.080	.081	.090	.081	.268	.030	.088	.731
(Impulsivity)	.106	.078	.170	.189	.076	.013	.447	.086	<.001
Interaction	−.044	.078	.578	.084	.076	.269	−.123	.077	.112
Marriage Experience (0 = lifetime singlehood, 1 = having at least one marriage)									
Childhood SES	.332	.135	.014	.298	.133	.026	.045	.147	.758
(Impulsivity)	−.038	.134	.779	.158	.133	.238	.780	.162	<.001
Interaction	−.103	.136	.451	.126	.132	.342	−.050	.143	.727
Annual Household Income									
Childhood SES	.114	.064	.078	.118	.063	.063	.032	.066	.063
(Impulsivity)	−.045	.064	.483	.157	.063	.013	.202	.066	.002
Interaction	−.057	.063	.364	.094	.062	.129	−.077	.054	.151
Subjective SES									
Childhood SES	.224	.063	<.001	.223	.061	<.001	.097	.061	.115
(Impulsivity)	−.060	.063	.346	.165	.061	.008	.365	.062	<.001
Interaction	.012	.061	.841	.089	.060	.140	.040	.050	.431
Life Satisfaction									
Childhood SES	.219	.063	<.001	.200	.062	.001	.067	.060	.266
(Impulsivity)	.024	.063	.702	−.000	.062	.994	.418	.061	<.001
Interaction	−.011	.061	.862	.142	.061	.020	.012	.050	.803

Note. Childhood SES and impulsivity were standardised for the set of these multiple regression analyses. Number of children was submitted to Poisson regression analyses, and marriage experience was submitted to logistic regression analyses. For the remaining dependent variables (i.e., annual household income, subjective SES, and life satisfaction), reported regression coefficients are standardized coefficients. For the shaded cell (the significant interaction effect), we conducted a simple slope test. The interaction was not consistent with the hypothesis (the slope is positive for low childhood SES group and negative for high childhood SES group).

Table S8

Results of a Series of Multiple Regression Analyses (Male Participants)

	Impulsivity Variable								
	Temporal Discounting			Risk Taking			Mini K		
	b	*se*	*p*	*b*	*se*	*p*	*b*	*se*	*p*
Number of Children									
Childhood SES	.185	.073	.011	.160	.074	.031	.087	.080	.277
(Impulsivity)	.034	.070	.622	.244	.070	<.001	.439	.074	<.001
Interaction	.019	.065	.773	.026	.071	.709	−.028	.063	.657
Marriage Experience (0 = lifetime singlehood, 1 = having at least one marriage)									
Childhood SES	.237	.114	.038	.208	.114	.068	.007	.126	.958
(Impulsivity)	.028	.113	.803	.163	.113	.147	.880	.149	<.001
Interaction	−.011	.103	.914	.112	.112	.317	.046	.127	.717
Annual Household Income									
Childhood SES	.088	.055	.113	.111	.055	.043	.039	.055	.480
(Impulsivity)	−.066	.056	.235	.142	.055	.010	.028	.057	<.001
Interaction	.077	.050	.124	.089	.053	.096	.036	.044	.416
Subjective SES									
Childhood SES	.219	.055	<.001	.238	.054	<.001	.141	.053	.008
(Impulsivity)	−.022	.055	.689	.147	.054	.006	.336	.055	<.001
Interaction	.069	.049	.164	.074	.053	.159	−.030	.043	.491
Life Satisfaction									
Childhood SES	.248	.055	<.001	.263	.054	<.001	.111	.050	.027
(Impulsivity)	−.040	.055	.471	.008	.054	.884	.482	.051	<.001
Interaction	−.007	.050	.887	.140	.053	.008	.030	.040	.445

Note. Childhood SES and impulsivity were standardised for the set of these multiple regression analyses. Number of children was submitted to Poisson regression analyses, and marriage experience was submitted to logistic regression analyses. For the remaining dependent variables (i.e., annual household income, subjective SES, and life satisfaction), reported regression coefficients are standardized coefficients. For the two shaded cells (one significant and one marginally significant interaction effects), we conducted a series of simple slope tests. Neither of the interactions was consistent with the hypothesis (the slope is positive for low childhood SES group and negative for high childhood SES group).

Table S9

Results of a Series of Multiple Regression Analyses (Female Participants)

	Impulsivity Variable								
	Temporal Discounting			Risk Taking			Mini K		
	b	*se*	*p*	*b*	*se*	*p*	*b*	*se*	*p*
Number of Children									
Childhood SES	.066	.063	.293	.027	.063	.666	−.048	.067	.476
(Impulsivity)	.049	.062	.426	.150	.060	.012	.360	.066	<.001
Interaction	−.114	.061	.063	.095	.056	.092	−.043	.063	.488
Marriage Experience (0 = lifetime singlehood, 1 = having at least one marriage)									
Childhood SES	.328	.121	.007	.325	.120	.007	.163	.137	.234
(Impulsivity)	−.129	.117	.270	.255	.125	.041	.741	.142	<.001
Interaction	−.124	.119	.301	.104	.122	.393	.116	.131	.376
Annual Household Income									
Childhood SES	.238	.053	<.001	.231	.053	<.001	.158	.055	.004
(Impulsivity)	−.089	.053	.095	−.024	.053	.655	.195	.054	<.001
Interaction	−.076	.052	.147	.058	.051	.257	−.106	.049	.033
Subjective SES									
Childhood SES	.313	.052	<.001	.302	.051	<.001	.023	.052	<.001
(Impulsivity)	.017	.052	.750	.172	.051	<.001	.295	.052	<.001
Interaction	.009	.051	.856	−.037	.049	.452	−.038	.047	.421
Life Satisfaction									
Childhood SES	.291	.052	<.001	.281	.052	<.001	.136	.049	.006
(Impulsivity)	.052	.052	.321	.073	.052	.161	.479	.049	−.001
Interaction	−.031	.051	.549	.065	.050	.197	−.044	.044	.322

Note. Childhood SES and impulsivity were standardised for the set of these multiple regression analyses. Number of children was submitted to Poisson regression analyses, and marriage experience was submitted to logistic regression analyses. For the remaining dependent variables (i.e., annual household income, subjective SES, and life satisfaction), reported regression coefficients are standardized coefficients. For the three shaded cells (one significant and two marginally significant interaction effects), we conducted a series of simple slope tests. Of the three, the marginally significant interaction between childhood SES and temporal discounting on number of children tended to be supportive of the life history hypothesis (the slope is positive and significant for low childhood SES group and negative but non-significant for high childhood SES group), while the other two interactions were not consistent with the hypothesis.

Table S10

Results of a Series of Planned Contrasts Using the Number of Marriages as the Dependent Variable

	Variable Used for Grouping								
	Temporal Discounting			Risk Taking			Mini K		
	b	*se*	*p*	*b*	*se*	*p*	*b*	*se*	*p*
All participants									
DV = Number of Marriage									
	*n*s = 173, 149, 190, and 158 for Groups 1, 2, 3, and 4			*n*s = 167, 160, 183, and 165 for Groups 1, 2, 3, and 4			*n*s = 185, 138, 145, and 197 for Groups 1, 2, 3		
Contrast 1	0.07	0.07	.342	−0.11	0.07	.110	**0.21**	0.07	.004
Contrast 2	−0.00	0.07	.993	−0.04	0.07	.526	***0.26***	0.07	.000
Contrast 3	0.13	0.10	.205	0.11	0.10	.256	0.04	0.10	.666
Four groups divided into upper/lower tertiles of childhood SES and impulsivity									
DV = Number of Marriage									
	*n*s = 86, 62, 85, and 82 for Groups 1, 2, 3, and 4			*n*s = 84, 66, 98, and 73 for Groups 1, 2, 3, and 4			*n*s = 93, 40, 55, and 102 for Groups 1, 2, 3, and 4		
Contrast 1	0.07	0.10	.468	−0.07	0.10	.489	0.24	0.12	.053
Contrast 2	−0.05	0.11	.625	−0.05	0.11	.661	***0.30***	0.11	.008
Contrast 3	**0.33**	0.15	.025	**0.38**	0.15	.009	0.21	0.17	.207
Only participants whose annual household income decreased from the last year									
DV = Number of Marriage									
	*n*s = 49, 54, 72, and 70 for Groups 1, 2, 3, and 4			*n*s = 59, 48, 80, and 61 for Groups 1, 2, 3, and 4			*n*s = 72, 36, 58, and 84 for Groups 1, 2, 3, and 4		
Contrast 1	0.07	0.12	.570	−0.08	0.12	.497	0.11	0.13	.379
Contrast 2	0.07	0.11	.529	−0.11	0.11	.306	***0.37***	0.11	<.001
Contrast 3	0.17	0.16	.293	0.11	0.16	.485	0.05	0.17	.753
Only male participants									
DV = Number of Marriage									
	*n*s = 88, 65, 94, and 80 for Groups 1, 2, 3, and 4			*n*s = 83, 73, 81, and 90 for Groups 1, 2, 3, and 4			*n*s = 85, 71, 76, and 93 for Groups 1, 2, 3, and 4		
Contrast 1	0.02	0.11	.886	−0.18	0.11	.102	**0.26**	0.12	.028
Contrast 2	0.04	0.11	.731	0.07	0.11	.521	***0.39***	0.12	<.001
Contrast 3	0.14	0.16	.368	0.13	0.16	.430	0.08	0.17	.622
Only female participants									
DV = Number of Marriage									
	*n*s = 89, 79, 83, and 89 for Groups 1, 2, 3, and 4			*n*s = 88, 82, 96, and 79 for Groups 1, 2, 3, and 4			*n*s = 101, 66, 68, and 103 for Groups 1, 2, 3, and 4		
Contrast 1	0.03	0.09	.714	−0.07	0.09	.398	0.13	0.09	.153
Contrast 2	−0.08	0.09	.371	−0.17	0.09	.059	0.15	0.09	.095
Contrast 3	0.12	0.13	.349	0.10	0.13	.418	0.04	0.13	.777

Note. Number of marriages was submitted to Poisson regression analyses. **Bold** coefficients indicate significant difference in the hypothesised direction. ***Bold italic*** coefficients indicate significant difference opposite to the hypothesised direction.
